# Influence of weeding methods on rhizosphere soil and root endophytic microbial communities in tea plants

**DOI:** 10.3389/fmicb.2024.1334711

**Published:** 2024-02-07

**Authors:** Yuxiao Yan, Conglian Wang, Renyuan Wan, Shuang Li, Yanfen Yang, Caiyou Lv, Yongmei Li, Guangrong Yang

**Affiliations:** ^1^College of Tea Science, Yunnan Agricultural University, Kunming, Yunnan, China; ^2^College of Resources and Environment, Yunnan Agricultural University, Kunming, Yunnan, China

**Keywords:** tea plant, polyethylene mulch, weeding, rhizosphere soil, endophytic microorganism

## Abstract

**Introduction:**

Polyethylene mulch is a kind of inorganic mulch widely used in agriculture. The effects of plastic mulch debris on the structure of plant soil and root growth have been fully studied, but their effects on endophytic microbial communities have not been explored to a large extent.

**Methods:**

In this study, High-throughput sequencing of bacterial 16S rRNA genes and fungal ITS region sequences were used to analyze microbial community structure and composition in rhizosphere soil and root endophytic of tea plant under three different weeding methods: polyethylene mulching, hand weeding and no weeding (CK).

**Results:**

The results showed that the weeding methods had no significant effect on the rhizosphere and root endophytic microbial abundance, but the rhizosphere bacterial structure covered by polyethylene mulch was significantly different than hand weeding and CK. The rhizosphere fungal diversity was also significantly higher than the other two analyzed treatments. The community abundance of rhizosphere microorganisms Acidobacteria, Candidatus Rokubacteria and *Aspergillus* covered by polyethylene mulch decreased significantly, whereas *Bradyrhizobium*, *Solirubrobacterales* and *Alphaproteobacteria* increased significantly. The abundance of bacteria *Ktedonobacter*, *Reticulibacter*, *Ktedonosporobacter* and *Dictyobacter* communities covered by polyethylene mulch was significantly changed, and the abundance of *Fusarium* and Nitrobacteraceae was significantly increased. Rhizosphere dominant bacteria were negatively correlated with soil available nitrogen content, while dominant fungi were significantly correlated with soil pH, total nitrogen and total potassium.

**Discussion:**

Polyethylene mulch forms an independent micro-ecological environment. At the same time, the soil nutrient environment was enriched by affecting the nitrogen cycle, and the composition of microbial community was affected. This study elucidated the effects of polyethylene mulch on soil microbial community in tea garden and provided a new theoretical understanding for weed management.

## 1 Introduction

Mulch is a protective covering around crops, designed to promote plant growth, control weed growth, increase crop yield, and control pests. In 2019, the use of agricultural plastic mulch in China reached 2.41 million tons. It is expected that China’s mulch coverage area will reach 23.4 million hm^2^ by 2025 (Qi R. et al., 2020). The most widely used non-degradable agricultural film in China is polyethylene (PE) agricultural film, followed by polypropylene (PP) (Li et al., 2021). Polyethylene mulch is the most popular and commonly used inorganic mulch film in the world because of its good effect and low price (Zhang et al., 2021). These mulches are very effective in the field because of their low cost, low replacement frequency, and high versatility (Manzano et al., 2019). Plastic mulch can improve runoff efficiency and rainwater collection, increase soil nutrient availability, and control crop diseases and pests (Wang et al., 2009; Chai et al., 2014).

It is often impossible to completely remove polyethylene mulch from fields after agricultural activities, which results in the presence of large amounts of macro and micro plastic mulch debris in the soil (Sarkar et al., 2018). At the same time, the aging and degradation causes plastic mulch fragments become microplastics and accumulate continuously in the soil (Qiang et al., 2023). The accumulation of such plastic residues in soil negatively affects crop production by affecting plant growth-promoting bacteria (PGPB), damaging soil structure, hindering root growth and development, and altering soil carbon concentration (Bai et al., 2015). Plastic mulch debris lead to uneven distribution of water and nitrate, thus affecting plant root growth. Plastic film debris can also inhibit the growth of soil microorganisms and animals (Zhang et al., 2019; Qi R. et al., 2020). In some countries, due to a lack of disposal options, farmers casually store these mulch after use, which subsequently leads to the dispersion of plastic mulch debris into the environment by water and wind erosion, causing contamination (Hayes et al., 2019).

Soil microorganisms play an important role in nutrient cycling and structural maintenance of agroecosystems (Nacke et al., 2011; Tian et al., 2015). Changes in soil physical and chemical properties during mulching can drive changes in microbial community (Qian et al., 2018), thus affecting microbial community structure and metabolic function (Djemiel et al., 2017; Dong et al., 2017). As the most abundant microbial group in soil, the interaction between bacteria and fungi is very common in soil (Jiao et al., 2022). For example, soil fungi may dominate the breakdown of refractory organic matter, such as lignin, and bacteria may symbiotically utilize fungal-derived substrates (Venkatalaxmi et al., 2004). Therefore, the structure and composition of bacterial and fungal communities in the soil were used to understand the effects of polyethylene mulch on agricultural production.

The tea plant [*Camellia sinensis* (L.) O. Kuntze] is evergreen, and its soil environment is different from other crops. However, studies of inorganic or organic mulch on soil microbial diversity and composition and soil fertility in tea gardens were rather limited. In this study, the diversity, community structure and composition of soil bacteria and fungi in rhizosphere soil and root endophytic microorganisms under 3 different weeding methods were investigated through field experiments. We hope it will provide theoretical information for the use of polyethylene mulch in tea gardens and for promoting the development of tea industry.

## 2 Materials and methods

### 2.1 Sampling time and sampling location

The sampling was April 2022, and the site was in Amuga Village, Nuofu Township, Lahu Autonomous County, Lancang. The highest altitude of the village was 1640 m above sea level (a.s.l), the lowest altitude was 900 m above sea level (a.s.l), the annual average temperature was 16.5 C, and the annual rainfall was 1673 mm. The information of sample collection sites is shown in Table 1. The tea gardens in the three plots are all located on the same slope, and the cultivated tea plant varieties were all local population species from Jingmai Mountain. The three sampling sites had different weeding methods. Sample site A was covered with polyethylene mulch, which was laid before December 2019 and covered for more than 2 years. Sample B was weeding by artificial tillage. The C sample site was managed without weeding. All the tea plantations were managed by the same person. Except for different weeding methods, all the cultivation measures were carried out simultaneously. Shade trees were planted in all the sample sites. Chemical fertilizer and pesticide were not applied. A total of 9 soil rhizosphere samples and 9 root samples were obtained under every weeding treatment repeated three times.

**TABLE 1:** Information on sampling sites of rhizosphere soil and roots of tea plants.

Site number	Longitude	Latitude	Altitude (m)	Air pressure(hpa)	Management pattern
A	E99°53′22″	N22°14′28″	1495.9	842.8	Polyethylene mulch weeding
B	E99°53′22″	N22°14′28″	1498.3	842.6	Hand weeding
C	E99°46’28″	N22°14′25″	1556.2	838.9	No weeding

### 2.2 Collection and treatment of rhizosphere soil and root samples

Before sampling, the mulch was removed from the sampling area to prevent surface organic matter from contaminating the soil layer. After that, leaf litter, weeds, stones and topsoil were appropriately removed, and tea tree roots were dug up from 20 cm soil depth. A sample of rhizosphere soil is obtained by gently brushing away the soil tightly adhered to the roots of the tea plant using a sterile soft-bristled paintbrush. After removing the rhizosphere soil, use sterile scissors to cut the tea plant roots with a diameter of 1–5 mm, put the collected root sample into a sterile bag, place it on ice, and transport it back to the laboratory. The obtained rhizosphere soil was evenly mixed and divided into two parts. One soil sample (about 20 g) was frozen in liquid nitrogen, transported to the laboratory on dry ice, and stored at −80°C for microbial metagenomic detection. The other portion was put into a sampling bag, dried naturally in the laboratory and screened 2 mm, 1 mm, 0.25 mm, 0.15 mm for the determination of soil pH value, available nitrogen (AN), available phosphorus (AP), available potassium (AK), organic matter (SOM), total nitrogen (TN), total phosphorus (TP), total potassium (TK).

The collected tea plant root samples were disinfected on the surface, washed with sterile water for 30 s, soaked in 75% ethanol for 2 mins, soaked with 2.5% NaClO for 5 mins, then transferred to 75% sterile ethanol for 30 s, and finally washed with sterile water for 3 times. Take the last clean sterile water 100 μL and apply it to the Potato Dextrose Agar (PDA) plate to check whether the plant surface is sterile. After washing, the samples were stored in a sterile bag at −80°C, and promptly sent to Shanghai Meiji Biomedical Technology Co., LTD., for microbial community diversity testing.

### 2.3 Determination of physicochemical properties of rhizosphere soil of tea plants

Soil pH value was determined by glass composite electrode method using 2.5:1 water and soil (Yang et al., 2020). Soil organic matter (SOM) content was measured by potassium dichromate capacity method-external heating method; Total nitrogen (TN) content was measured by Kjeldahl nitrogen determination after concentrated sulfuric acid and hydrogen peroxide elimination; Total phosphorous (TP) content was determined by molybdenum-antimony resistance colorimetric method after digestion of concentrated sulfuric acid and hydrogen peroxide; Total potassium (TK) content was determined by sodium hydroxide melt-flame spectrophotometry; Available nitrogen (AN) content was measured using alkalescent diffusion method of 0.5 mol L^–1^ NaOH diffusion method; Available phosphorus (Olsen-P, AP) content was determined through molybdate blue colorimetric method after the extract of 0.5 mol L^–1^ NaHCO_3_ solution; Available potassium (AK) was adopted by ammonium acetate extraction-flame spectrophotometry.

### 2.4 DNA extraction, library construction, and metagenomic sequencing

Total genomic DNA was extracted from soil and roots samples using the Mag-Bind^®^ Soil DNA Kit (Omega Bio-tek, Norcross, GA, U.S.) according to manufacturer’s instructions. Concentration and purity of extracted DNA was determined with TBS-380 and NanoDrop2000, respectively. DNA extract quality was checked on 1% agarose gel. The PCR primers 338F (5′- ACTCCTACGGGAGGCAGCAG-3′) and 806R (5′- GGACTACHVGGGTWTCTAAT -3′) were used to amplify bacterial 16S rRNA, and ITS1F (5′- GCTGCGTTCTTCATCGATGC -3′) and ITS2R (5′- GCTGCGTTCTTCATCGATGC-3′) were used to amplify fungal ITS. DNA extract was fragmented to an average size of about 400 bp using Covaris M220 (Gene Company Limited, China) for paired-end library construction. Paired-end library was constructed using NEXTFLEX Rapid DNA-Seq (Bioo Scientific, Austin, TX, USA). Adapters containing the full complement of sequencing primer hybridization sites were ligated to the blunt-end of fragments. Paired-end sequencing was performed on Illumina NovaSeq (Illumina Inc., San Diego, CA, USA) at Majorbio Bio-Pharm Technology Co., Ltd. (Shanghai, China) using NovaSeq 6000 S4 Reagent Kit v1.5 (300 cycles) according to the manufacturer’s instructions.^1^ Sequence data associated with this project have been deposited in the NCBI Short Read Archive database.

### 2.5 Sequence quality control and genome assembly

Briefly, the paired-end Illumina reads were trimmed of adaptors, and low-quality reads (length < 50 bp or with a quality value < 20 or having N bases) were removed by fastp (Chen et al., 2018) (version 0.20.0).^2^ Reads were compared with host DNA sequences by software BWA (Chong et al., 2013) (version 0.7.9a),^3^ and contaminated reads with high similarity were removed.

Metagenomics data were assembled using MEGAHIT (Li et al., 2015) (version 1.1.2),^4^ which makes use of succinct de Bruijn graphs. Contigs with with a length ≥ 300 bp were selected as the final assembling result, and then the contigs were used for further gene prediction and annotation.

### 2.6 Gene prediction, taxonomy, and functional annotation

Open reading frames (ORFs) from each assembled contig were predicted using Prodigal (Hyatt et al., 2015).^5^ The predicted ORFs with a length ≥ 100 bp were retrieved and translated into amino acid sequences using the NCBI translation table.^6^

A non-redundant gene catalog was constructed using CD-HIT (Fu et al., 2012) (version 4.6.1)^7^ with 90% sequence identity and 90% coverage. High-quality reads were aligned to the non-redundant gene catalogs to calculate gene abundance with 95% identity using SOAP aligner (Li et al., 2008) (version 2.21).^8^

Representative sequences of non-redundant gene catalog were aligned to NR database with an e-value cutoff of 1^e–5^ using Diamond (Buchfink et al., 2015) (version 0.8.35)^9^ for taxonomic annotations.

### 2.7 Statistical analysis

Data means and standard deviations were computed and statistically analyzed using SPSS version 16 (SPSS, Inc., Chicago, IL, United States). The Shapiro-Wilk test was used to check normality of the data, and pairwise comparisons were made between the study groups with the non-parametric test Kruskal-Wallis one-way analysis of variance, which compares means between groups. False discovery rate (FDR) corrected p-values were taken into account to consider significant results. Using mothur (version v. 1.30.1)^10^ to analyze the alpha diversity of microbial communities, the index assessed an OTU similarity level of 97% (0.97). R version 2.1.3 was used to conduct principal coordinate analysis (PCoA) to condense the original variables’ dimensions based on Bray-Curtis distances. Using LEfSe,^11^ linear discriminant analysis (LDA) was performed on samples according to different grouping conditions according to taxonomic composition, and species that had significant differential effects on sample classification were identified. An LDA score > 2.0 and *p* ≤ 0.05 were used to filter indicator genera that were considered ‘extremely enriched’. Using R “pheatmap,” a Spearman correlation heatmap was generated to examine the relationship between the structures of the rhizosphere soil microbial community and the factors affecting the soil environment. The data were analyzed on the free online platform of Majorbio Cloud Platform.^12^

## 3 Result

### 3.1 The diversity of soil bacterial and fungal community

We calculated the indices of alpha diversity such as Chao1, ACE, Simpson, and Shannon to quantify the diversity and richness of the rhizosphere soil and root endophytic microbial community among the three treatments. The coverage indexes of 9 rhizosphere soil samples and 9 root samples were all greater than 0.94, indicating that the sequencing capability was acceptable (Table 2). There was no significant difference in the richness estimator and diversity index of the root endophytic bacterial and fungal communities among different treatments. For rhizosphere soil bacterial communities, there was no significant difference in the richness of the three treatment groups, while the diversity index of AS was significantly higher than BS and CS. For rhizosphere soil fungal communities, the richness of BS was significantly lower than AS and CS, and the Shannon index of CS was significantly higher than AS and BS.

**TABLE 2:** The diversity indices of soil bacterial and fungal communities.

Sample	Diversity index		Richness estimator		Coverage(%)
	**Shannon**	**Simpson**	**Ace**	**Chao1**	
**Bacteria**					
AR	6.20 ± 0.19a	0.020 ± 0.002a	12035.78 ± 2780.74a	12218.69 ± 2857.26a	99.57
BR	6.36 ± 0.57a	0.010 ± 0.007a	11659.32 ± 1458.62a	11813.76 ± 1387.73a	99.86
CR	6.03 ± 0.22a	0.022 ± 0.007a	11305.14 ± 2247.35a	11444.52 ± 2389.29a	99.65
AS	5.94 ± 0.22a	0.018 ± 0.001b	24157.44 ± 121.82a	24371.64 ± 126.21a	99.97
BS	5.69 ± 0.08b	0.027 ± 0.003a	24260.45 ± 438.21a	24438.99 ± 463.17a	99.98
CS	5.58 ± 0.07b	0.031 ± 0.003a	24465.40 ± 300.48a	24647.50 ± 317.99a	99.98
**Fungi**					
AR	3.31 ± 1.22a	0.188 ± 0.154a	417.92 ± 201.32a	394.37 ± 172.56a	95.54
BR	4.67 ± 0.53a	0.036 ± 0.025a	536.52 ± 338.10a	546.32 ± 330.28a	98.46
CR	4.72 ± 0.59a	0.014 ± 0.004a	322.85 ± 221.21a	322.07 ± 227.52a	94.97
AS	4.81 ± 0.04ab	0.030 ± 0.007a	459.18 ± 11.79a	463.31 ± 18.17a	97.71
BS	4.68 ± 0.29b	0.029 ± 0.010a	356.22 ± 55.62b	347.23 ± 63.66b	94.89
CS	5.07 ± 0.07a	0.017 ± 0.002a	464.21 ± 7.01a	462.06 ± 6.72a	97.93

Data are presented as mean values ± SE (Standard error). Different letters indicate significant difference among different treatment, according to statistics analysis. Using SPSS statistics 26 with Duncan’s multiple range test (*P* ≤ 0.05). A: polyethylene mulch weeding. B: hand weeding. C: no weeding. S: rhizosphere soil. R: roots.

**FIGURE 1:**
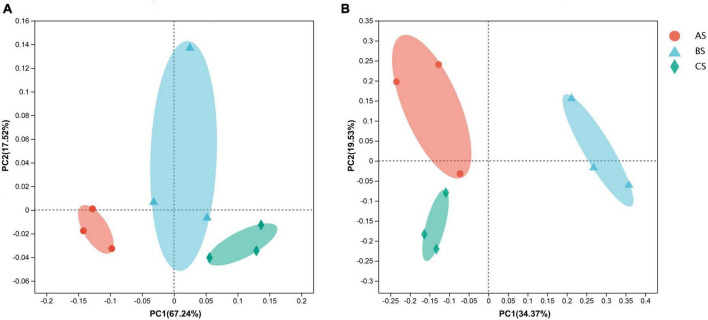
The principal coordinate analysis (PCoA) based on Bray–Curtis distance in microbial communities in rhizosphere soil and roots under different weeding methods. **(A)** The distribution of bacterial communities in rhizosphere soil under different weeding methods. **(B)** The distribution of fungal communities in rhizosphere soil under different weeding methods. A: polyethylene mulch weeding. B: hand weeding. C: no weeding.

PCoA of the 16S rRNA sequencing data revealed that the bacterial community 84.76% of the variation accounted for by PCoA1 and PCoA2 (Figure 1A), while PCoA of the ITS region sequencing revealed that 53.9% of the variation accounted for by PCoA1 and PCoA2 in the fungal community composition (Figure 1B). The different replicates from each treatment cluster together. According to the 16S rRNA data, AS was separated from the other two groups on the X-axis. Based on ITS gene sequencing, BS clustered on the positive side of the X-axis and CS clustered on the negative side of the Y-axis.

In the root endophytic microbial community, PCoA of the 16S rRNA sequencing data showed that the bacterial community 63.62% of the variation accounted for by PCoA1 and PCoA2 (Supplementary Figure 1A), while PCoA of the ITS region sequencing showed that 50.5% of the variation accounted for by PCoA1 and PCoA2 in the fungal community composition (Supplementary Figure 1B). According to the 16S rRNA data and ITS gene sequencing, the different replicates from each treatment didn’t cluster together.

### 3.2 Composition of rhizosphere soil and root endophytic microbial community

**FIGURE 2:**
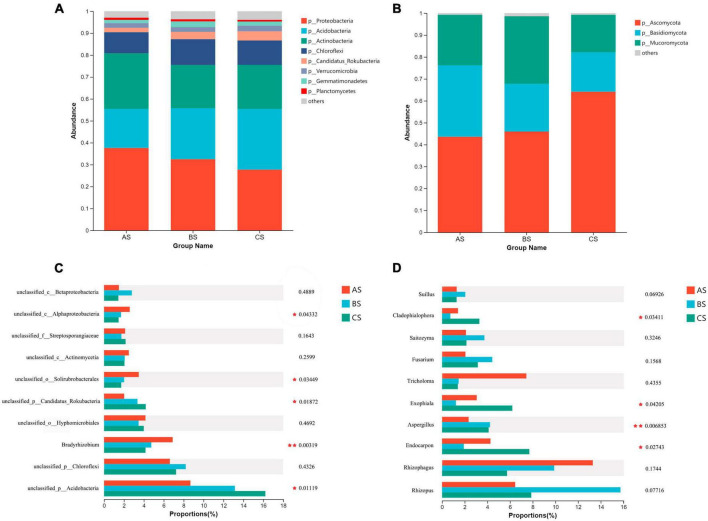
Relative abundance of main microbial communities in rhizosphere soil under different weeding methods. **(A)** Relative abundance of dominant bacterial communities at the phylum level. **(B)** Relative abundance of dominant fungal communities at the phylum level. **(C)** Relative abundance of the top 10 bacterial communities at genus level. **(D)** Relative abundance of the top 10 fungal communities at genus level.

**FIGURE 3:**
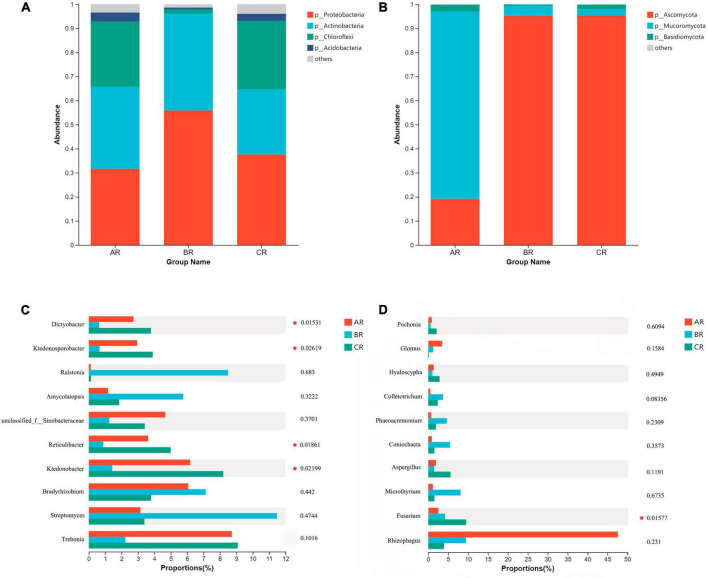
Relative abundance of main root endophytic microbial communities under different weeding methods. **(A)** Relative abundance of dominant bacterial communities at the phylum level. **(B)** Relative abundance of dominant fungal communities at the phylum level. **(C)** Relative abundance of the top 10 bacterial communities at genus level. **(D)** Relative abundance of the top 10 fungal communities at genus level.

We compared the composition of bacterial and fungal community structures at phylum and genus level, rhizosphere soil and root system under different weeding methods. The dominant major phyla in the rhizosphere soil and root endophytic bacterial community are *Proteobacteria*, *Acidobacteria*, *Actinobacteria*, and *Chloroflexi* (Figures 2A, 3A). *Bradyrhizobium*, *Ktedonobacter*, *Reticulibacter*, *Ktedonosporter* and *Dictyobacter* showed significant differences at genus level (Figures 2C, 3C). Basidiomycota, Ascomycota and Mucoromycota were the three main fungal phyla in the rhizosphere soil and root endophytic (Figures 2B, 3B). *Fusarium*, *Endocarpon*, *Aspergillus*, *Exophiala* and *Cladophialophora* showed significant differences at genus level (Figures 2D, 3D).

**FIGURE 4:**
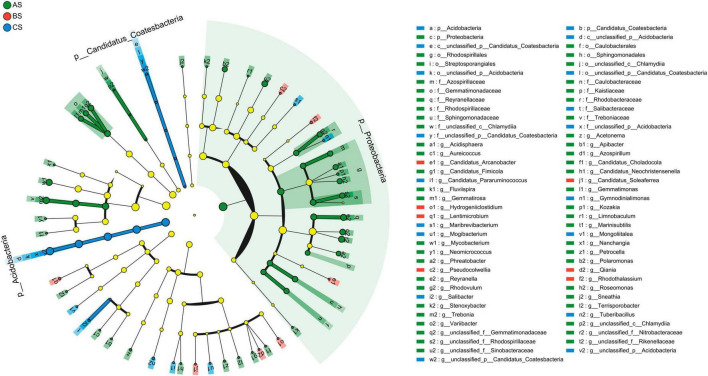
Linear discriminant analysis (LDA) effect size analysis of the evolutionary branch diagram in rhizosphere soil bacterial communities. From the inside to the outside in the figure was the classification of phylum to genus. Among them, the diameter of different dots was significantly positively correlated with species abundance.

LEfSe was used to identify bacteria and fungi with significant abundance differences in rhizosphere soil under three different weeding methods. Statistical analysis from phylum to genus level is performed in cladograms, and LDA > 2.0. Proteobacteria was the most abundant bacterial phylum, followed by Acidobacteria and Candidatus_Coatesbacteria (Figure 4). Ascomycota was the most abundant fungi phylum, followed by Mucoromycota and Basidiomycota (Supplementary Figure 4). In rhizosphere soil bacterial communities, there are 9 groups of bacteria in AS that are significantly enriched, including Treboniaceae (family to genus), Gemmatimonadaceae (family to genus), Caulobacterales (family to genus), Reyranellaceae (family to genus), Rhodobacteraceae (family to genus), Rhodospirillales (order to genus), Azospirillaceae (family to genus), Rhodospirillaceae (family to genus), Sphingomonadales (family to genus). Salibacteraceae (order to genus) were significantly enriched in CS (Supplementary Figure 3). In the rhizosphere soil fungal communities, two groups of fungi were significantly enriched in BS: Mucoromycetes (class to genus) and Sympoventuriaceae (family to genus). Three groups of fungi were significantly enriched in CS: Eurotiomycetes (class to genus), Herpotrichiellaceae (family to genus), and Verrucariales (order to genus) (Supplementary Figure 4). In the bacterial community, AS had significantly more biomarkers than the other two groups.

### 3.3 Relationships between the dominance phyla of microbial communities and rhizosphere soil physicochemical characteristics

Various physicochemical characteristics of rhizosphere soil samples collected from 3 different weeding methods were analyzed (Table 3). The TP content of the polyethylene mulch treatment group was significantly higher than hand weeding treatment group but lower than the control group, and the AN content was significantly higher than the other two treatment groups.

**TABLE 3:** Soil physicochemical characteristics in different weeding pattern.

	pH	SOM(g/kg)	TK(g/kg)	TP(g/kg)	TN(g/kg)	AN(mg/kg)	AP(mg/kg)	AK(mg/kg)
A	5.07 ± 0.07a	72.77 ± 11.33a	13.11 ± 3.36a	1.84 ± 0.30ab	2.38 ± 0.30a	233.62 ± 17.72a	7.96 ± 3.84a	360 ± 135.77a
B	5.16 ± 0.12a	52.35 ± 8.81a	15.78 ± 1.54a	1.59 ± 0.09b	2.33 ± 0.50a	112.78 ± 41.63b	4.26 ± 1.07a	308.89 ± 115.49a
C	5.10 ± 0.06a	70.81 ± 10.56a	12.22 ± 0.77a	2.07 ± 0.11a	3.05 ± 0.26a	109.04 ± 9.48b	5.52 ± 2.39a	243.33 ± 54.88a
*P*-value	0.464	0.929	0.257	0.051	0.179	0.037	0.074	0.299

Data are presented as mean values ± SE (Standard error). Different letters indicate significant difference among different treatment, according to statistics analysis. Using SPSS statistics 26 with Duncan’s multiple range test (*P* ≤ 0.05). A: polyethylene mulch weeding. B: hand weeding. C: no weeding.

**FIGURE 5:**
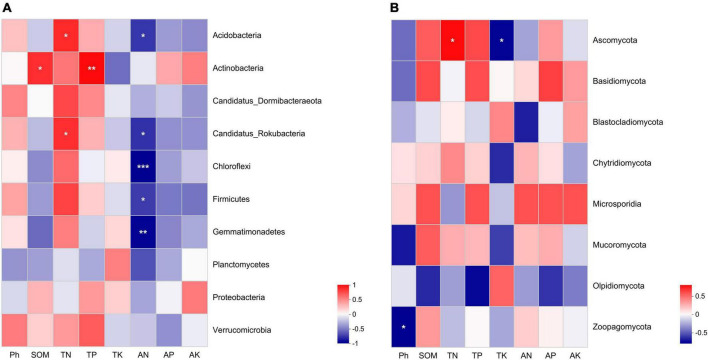
The spearman correlation heatmap between soil physicochemical characteristics and microbial communities in soils under different weeding methods. **(A)** The relationships between soil physicochemical characteristics and bacterial communities. **(B)** The relationships between soil physicochemical characteristics and fungal communities. “*” indicates a significant correlation at the *P* < 0.05 level; “**” indicates a very significant correlation at the *P* < 0.01 level; “***”indicates a very significant correlation at the *P* < 0.001 level.

We used spearman correlation heatmap to analyze the relationship between microbial dominance phyla and soil nutrient content and chemical properties. In the bacterial communities, soil TN was significantly positively correlated with Acidobacteria and Candidatus Rokubacteria. SOM and TP in soil were positively correlated with Actinobacteria. Soil AN was significantly negatively correlated with Acidobacteria, Candidatus Rokubacteria, Chloroflexi, Firmicutes and Gemmatimonadetes (Figure 5A). In fungal communities, soil pH was significantly negatively correlated with Zoopagomycota. Ascomycota was positively correlated with TN, but negatively correlated with TK (Figure 5B). The contents of soil AN were the main physicochemical factors affecting bacterial community structure.

## 4 Discussion

For a long time, the general effect of using polyethylene mulch in agricultural production on soil has been fully recognized. However, in the production of tea garden, the influence of polyethylene film on the composition of rhizosphere soil and root endophytic microorganisms and on the physical and chemical properties of tea garden soil is far from enough. In this study, to reveal the effects of polyethylene mulch on microbial community structure and soil physicochemical properties of tea plantation, 16S rRNA and ITS were used to analyze the diversity and community structure of bacteria and fungi in tea plants under three different weeding methods, polyethylene mulch, hand weeding and control, respectively. The results showed that polyethylene mulch could change microbial diversity and community structure of rhizosphere soil and root system in tea plants.

Appropriate microbial community structure and rich diversity play a key role in maintaining ecosystem sustainability and productivity (Zhao et al., 2014). Studies have shown that mulch alters the composition of microbial communities. Plastic mulch debris increased the total abundance of bacterial communities, but decreased their diversity and uniformity (Liu et al., 2022). However, polyethylene mulch has little effect on root endophytic microbial diversity in tea plants. In this study, polyethylene mulch significantly increased the diversity of rhizosphere soil and root endophytic bacteria, but significantly decreased the diversity of root endophytic fungi (Table 1).

On the other hand, we analyzed the difference of microbial community structure under different weeding methods by PCoA. Different weeding methods had an effect on the structure and composition of rhizosphere soil microbial community but had no effect on root endophytic microbial community. Therefore, under different weeding methods, rhizosphere soil and root system of tea garden constructed relatively independent micro-ecological environment. Meanwhile, polyethylene mulch treatment could obtain more abundant micro-ecological environment.

The microbial community structure was affected by different weeding methods. In this study, the dominant bacteria in rhizosphere soil and rhizosphere were Proteobacteria, Acidobacteria, Actinobacteria and Chloroflexi (Figure 2A). Proteobacteria is a very important phylum in soil. They have the ability to fix atmospheric nitrogen and decompose organic matter, and play a crucial role in nutrient cycling and soil fertility (Veerasamy et al., 2023). Most Actinobacteria are saprophytic and have a wide range of functions, promoting the decomposition of organic matter and the ability to decompose complex substrates, thus giving them a competitive advantage over other bacteria (Kamau et al., 2008; Nemergut et al., 2010). Acidobacteria has the ability to degrade cellulose and lignin from plant residues and is a common acidophilic bacteria in soil (Huang et al., 2015). We found that the relative abundance of Proteobacteria and Actinobacteria in the soil under the treatment of polyethylene mulch was significantly higher than the other two groups (Supplementary Figure 2), indicating that the treatment of polyethylene mulch provided a more abundant nutrient environment for the soil. However, the relative abundance of Acidobacteria is significantly lower than the other two groups. This may be because the polyethylene mulch physically prevents the entry of dead leaves and weeds into the soil, leading to a decrease in its relative abundance.

In addition, we also pay attention to the significant differences of Nitrobacteraceae at the soil family level. Polyethylene mulch debris were able to provide sufficient colonizing space for microorganisms, allowing Nitrobacteraceae to accumulate on them, and showed significant differences from the other groups (Supplementary Figure 5). Moreover, *Bradyrhizobium* also showed significant differences at rhizosphere soil genus level (Figure 2C). *Bradyrhizobium* is a gram-negative bacterium that widely exists in soil and plays an important role. Previous studies have shown that organisms from this genus play a crucial role in nitrogen fixation (Shah and Subramaniam, 2018). The significant enrichment of two groups microorganisms under polyethylene mulch treatment indicated that polyethylene mulch could enrich bacteria to participate in the nitrogen cycle of tea garden soil and promote the biochemical cycle of tea garden soil.

Studies have shown that polyethylene mulch can significantly affect the microbial community structure (Qi Y. et al., 2020), and soil microbe-mediated nutrient cycling can affect the physical and chemical conditions of soil dependent on plant growth and development (Qiang et al., 2023). In the current study on tea gardens, the content of AN in soil covered by polyethylene mulch was significantly higher than the other two groups (Table 3), which may be related to the significant enrichment of Nitrobacteraceae in the polyethylene mulch group. Nitrobacteraceae promoted the conversion of nitrite to nitrate in soil through nitrification to increase the content of AN in soil (Lücker et al., 2010). Spearman correlation heatmap analysis showed that the content of AN in soil was significantly negatively correlated with Acidobacteria, Candidatus Rokubacteria, Chloroflexi, Firmicutes and Gemmatimonadetes (Figure 5A). Polyethylene mulch changes the physical and chemical conditions of soil by changing the nutrient element circulation mediated by soil microorganisms, and then affects the community composition of soil microorganisms, which affects the growth and development of tea plants.

Chloroflexi shows a low-nutrient lifestyle and has a low growth rate under conditions of high nutrient utilization, which is an important green pigment phylum (Tardy et al., 2015). At the level of rhizosphere soil phyla, the relative abundance of Chloroflexi treated with polyethylene mulch was lower than the other two groups. But at the level of endophytic microbial phylum level, the relative abundance of Chloroflexi was significantly lower than CK but higher than the hand weeding group. This showed the same trend as the genus level (*Ktedonobacter*, *Reticulibacter*, *Ktedonosporobacter*, *Dictyobacter* all belong to Chloroflexi) (Figure 3C; Supplementary Figure 3). This showed that polyethylene mulch in the soil showed lower nutrient use efficiency than hand weeding treatment, and this effect was passed on to the roots.

In this study, the dominant fungal phyla in rhizosphere soil and root endophytic microbial communities were Ascomycota, Basidiomycota and Mucoromycota. As a major fungal decomposer in soil ecosystems (Wang et al., 2013), Ascomycota plays an invaluable role in the recycling of plant residues (Zhou et al., 2016). Under anaerobic conditions, Basidiomycota can help degrade lignin (Boer et al., 2005). The results showed that the relative abundance of Ascomycota in the polyethylene mulch treatment group was lower than the other two groups, and the relative abundance of Basidiomycota was higher than the other two groups. This indicated that the polyethylene mulch physically isolated the entry of litter into the soil and provided anaerobic conditions for the growth of Basidiomycota. This may be related to the long-term use of polyethylene mulch, resulting in soil compaction and poor air flow (Zhao et al., 2022).

## 5 Conclusion

In this study, high-throughput sequencing was used to study the effects of polyethylene mulch on microbial diversity and community structure in rhizosphere soil and root endophyte of tea plants. The results showed that the influence of different weeding methods on the structure and diversity of rhizosphere soil microbial community was greater than that of root. Polyethylene mulch treatment formed an independent micro-ecological environment. At the same time, the relative abundance of Proteobacteria, Actinobacteria, Nitrobacteraceae and *Bradyrhizobium* was increased, which enriched the soil nutrient environment and affected the composition of microbial community by affecting the nitrogen cycle.

## Data availability statement

All data used in this analysis are available in https://www.ncbi.nlm.nih.gov/. The associated SRA for all samples is PRJNA1053508. The SRA numbers are SAMN38860667, SAMN38860668, SAMN38860669, SAMN38860670, SAMN38860671, SAMN 38860672, SAMN38860673, SAMN38860674, SAMN38860 675, SAMN38860676, SAMN38860677, SAMN38860678, SAMN 38860679, SAMN38860680, SAMN38860681, SAMN38860682, SAMN38860683, and SAMN38860684, respectively.

## Author contributions

YXY: Data curation, Investigation, Software, Visualization, Writing–original draft. CW: Writing–original draft. RW: Writing–original draft.YFY: Writing–original draft. SL: Conceptualization, Formal Analysis, Methodology, Writing–original draft. CL: Funding acquisition, Resources, Writing–review and editing. YL: Writing–review and editing. GY: Resources, Supervision, Validation, Writing–review and editing.

## Footnotes

1
www.illumina.com

2
https://github.com/OpenGene/fastp

3
http://bio-bwa.sourceforge.net

4
https://github.com/voutcn/megahit

5
https://github.com/hyattpd/Prodigal

6
http://www.ncbi.nlm.nih.gov/Taxonomy/taxonomyhome.html/index.cgi?chapter=tgencodes#SG1

7
http://www.bioinformatics.org/cd-hit/

8
https://github.com/ShujiaHuang/SOAPaligner

9
https://github.com/bbuchfink/diamond

10
https://mothur.org/wiki/calculators/

11
http://galaxy.biobakery.org/

12
www.majorbio.com
