# Supplementary figures and images for: Influence of weeding methods on rhizosphere soil and root endophytic microbial communities in tea plants
Source: Front Microbiol. 2024 Feb 7;15:1334711. doi: 10.3389/fmicb.2024.1334711 (PMC10879617; doi:10.3389/fmicb.2024.1334711)

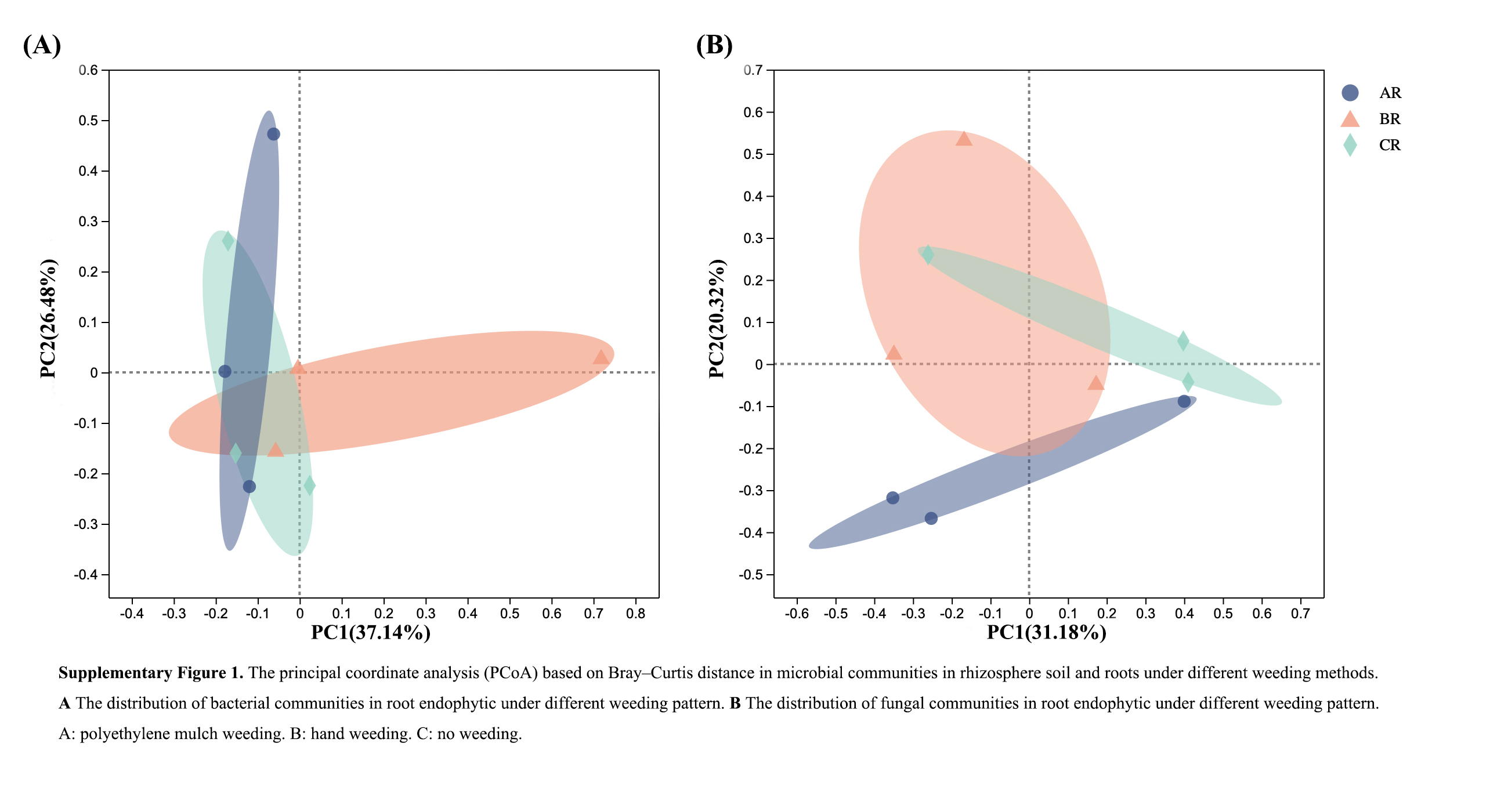

Supplement: Supplementary file 1 [file Image_1.JPEG]

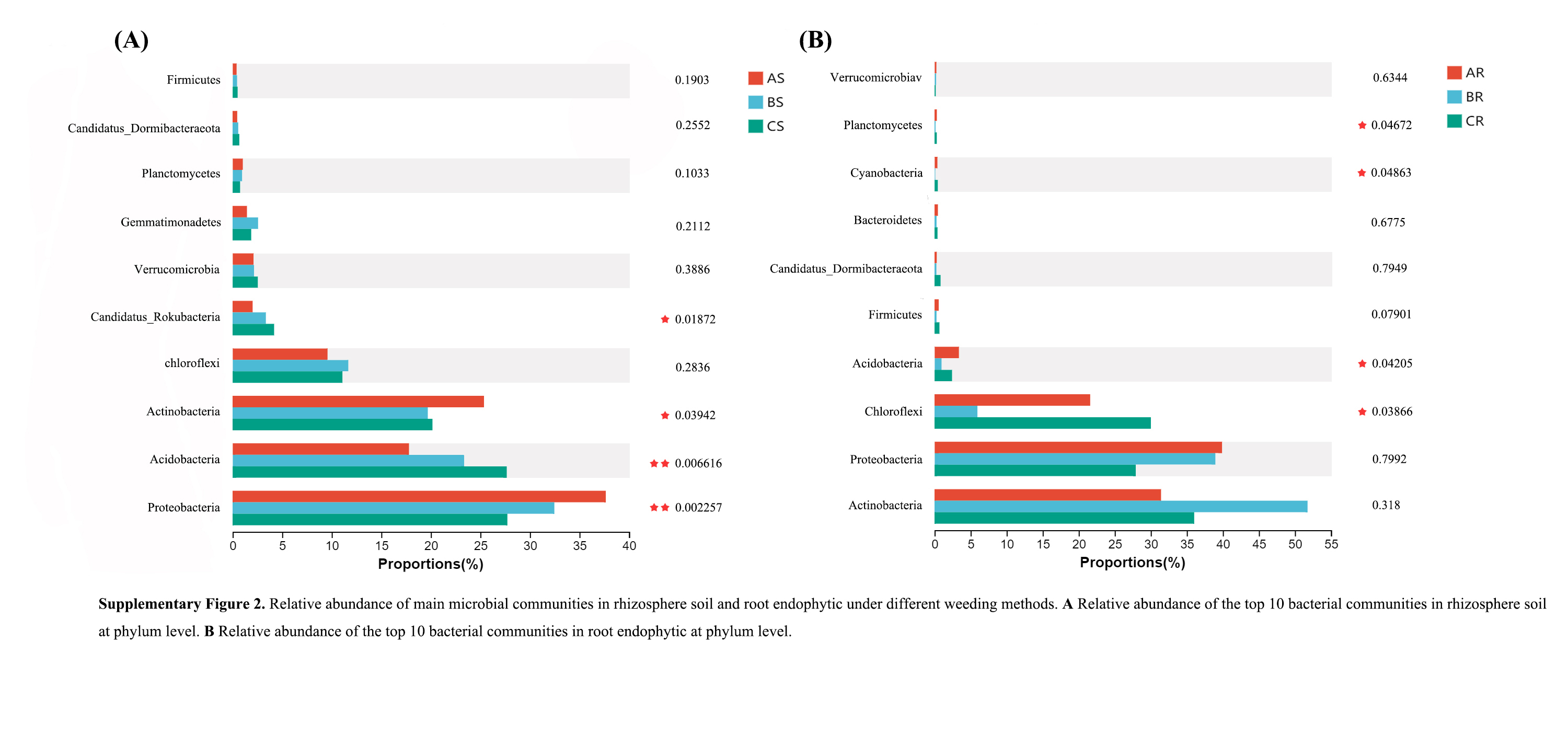

Supplement: Supplementary file 2 [file Image_2.JPEG]

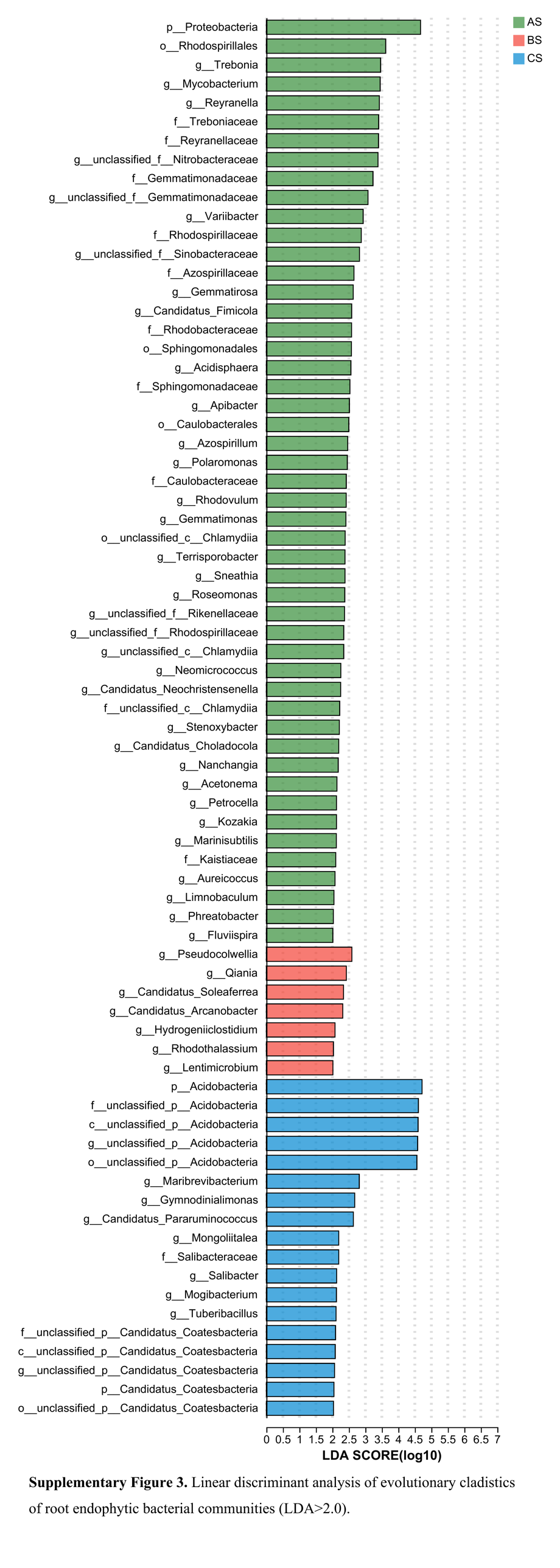

Supplement: Supplementary file 3 [file Image_3.JPEG]

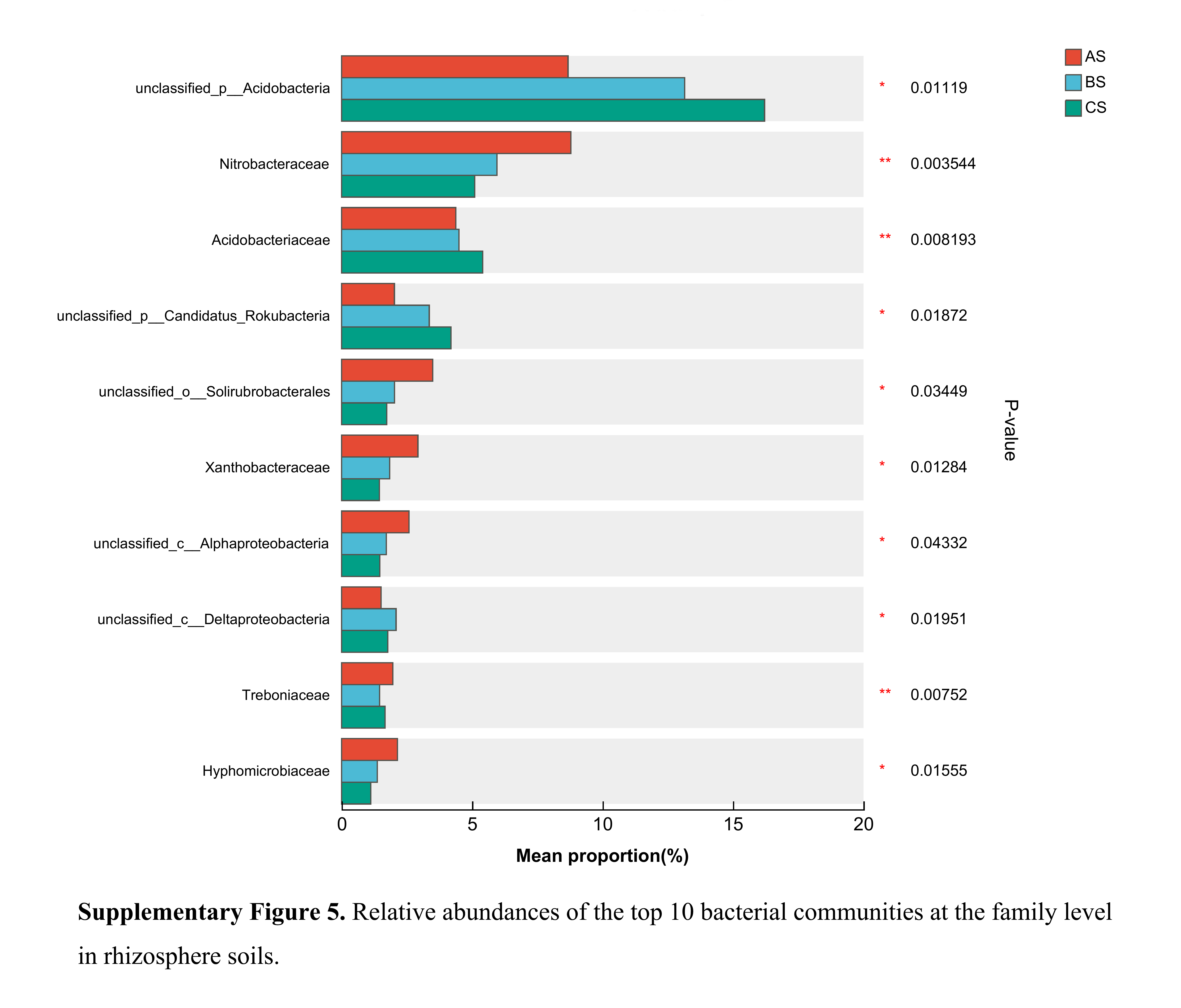

Supplement: Supplementary file 5 [file Image_5.JPEG]
